# Prediction of cardiovascular disease risk in women and individuals with polycystic ovary syndrome using the American Heart Association PREVENT model: A long-term population-based cohort study

**DOI:** 10.1016/j.ajpc.2026.101408

**Published:** 2026-01-03

**Authors:** Parham Heidari, Ramin Farrokhi, Faegheh Firouzi, Yasamin Zivari, Fereidoun Azizi, Fahimeh Ramezani Tehrani, Samira Behboudi-Gandevani

**Affiliations:** a Reproductive Endocrinology Research Center, Research Institute for Endocrine Sciences, Shahid Beheshti University of Medical Sciences, Tehran, Iran; b Department of Epidemiology and Biostatistics, School of Public Health, Tehran University of Medical Sciences, Tehran, Iran; c Endocrine Research Center, Research Institute for Endocrine Sciences, Shahid Beheshti University of Medical Sciences, Tehran, Iran; d Faculty of Nursing and Health Sciences, Nord University, Bodø, Norway; e Foundation for Research & Education Excellence, Vestavia Hills, Al, USA

**Keywords:** Cardiovascular disease, Polycystic ovary syndrome (PCOS), Prediction, PREVENT risk model, Tehran lipid and glucose study (TLGS)

## Abstract

**Background:**

This study aimed to assess the performance of the PREVENT risk model, in terms of discrimination and calibration in the overall female sample representative of the Iranian general population, across age groups, and among women with PCOS.

**Methods:**

In this population-based prospective study, we used data from phases 3–7 of the Tehran Lipid and Glucose Study (2006–2021). A total of 3983 women aged 30–79 years without baseline CVD and with complete data for the PREVENT risk score were included, of whom 2117 had known PCOS or isolated PCOS phenotypes status. The PREVENT risk model was applied to estimate 10-year CVD risk. The primary outcome was incident CVD, which was defined as a composite of fatal and nonfatal atherosclerotic cardiovascular disease (ASCVD) and heart failure (HF), identified follow-ups. Model discrimination was evaluated using Harrell’s C-statistics and time-dependent AUC, and calibration was assessed by comparing predicted and observed 10-year CVD risks in general female population, by age group, and by PCOS status.

**Results:**

Among 3983 women (median follow-up 12.2 years; mean age 47.6 years; mean BMI 28.6 kg/m²), 911 had confirmed PCOS/Isolated-PCOS phenotypes. In the overall population, the PREVENT risk model demonstrated good discrimination (C-statistic 0.84, 95 % CI: 0.82–0.86; AUC 0.80) and satisfactory calibration in lower- and mid-risk deciles, with some underestimation at the highest risk deciles. Discrimination declined with increasing age, performing best in women aged 30–44 years (C-statistic 0.82, AUC 0.83). Stratified by PCOS status, the model maintained good discrimination in women with PCOS/isolated PCOS phenotypes (C-statistic 0.79, AUC 0.81) and non-PCOS/ non-isolated PCOS phenotypes controls (C-statistic 0.85, AUC 0.81), with calibration remaining satisfactory across subgroups, though high-risk quartiles in older women were slightly underestimated.

**Conclusions:**

In this long-term, population-based cohort of Iranian women, the PREVENT risk model showed good discrimination and acceptable calibration for predicting 10-year CVD risk in both the general population and women with PCOS/Isolated-PCOS phenotypes. Performance was highest in women under 55 years, with some underestimation of risk in older or high-risk individuals.

## Graphical abstract

**Figure:**
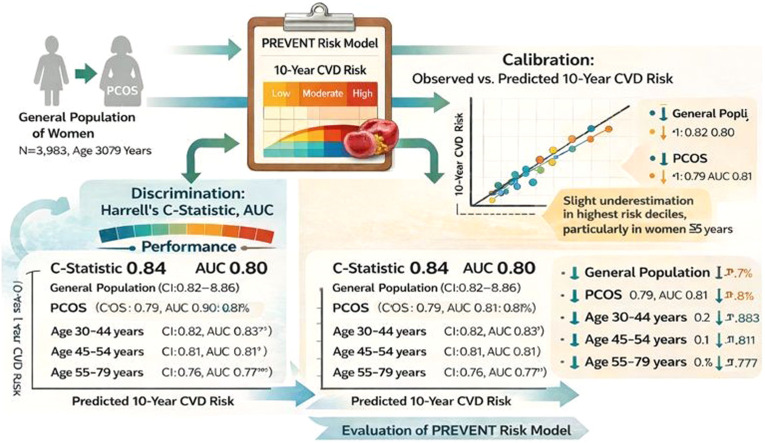

## 1 Introduction

Polycystic ovary syndrome (PCOS) is one of the most common endocrine disorders in reproductive-age women [1,2]. Beyond its role in reproductive dysfunction, PCOS is increasingly recognized as a systemic metabolic condition with significant implications for cardiometabolic health [3]. Several peer-reviewed studies confirm that PCOS is linked to an elevated risk of cardiovascular disease (CVD) through several pathophysiological mechanisms [4]. These mechanisms involve well-defined traditional CV risk factors, including insulin resistance (IR) and dyslipidemia [[5], [6], [7], [8]], alongside non-traditional risk factors such as hyperandrogenism and chronic inflammation [[9], [10], [11], [12]]. It is suggested that the synergistic effects of these traditional and non-traditional factors collectively contribute to the elevated cardiovascular risk observed in individuals with PCOS [4]. Additionally, emerging evidence suggests that renal dysfunction contributes significantly to the elevated CV risk observed in women with PCOS [13,14]. Studies have shown higher urinary albumin-to-creatinine ratio (UACR), IR, hypertension, metabolic comorbidities in this population, all of which are linked to impaired renal function and CVD [[15], [16], [17], [18]].

However, CV risk assessment is necessary for effective CVD prevention intervention, especially in high-risk individuals, such as PCOS. Traditional models, such as the Framingham Risk Score (FRS), the Pooled Cohort Equations, and Systematic Coronary Risk Evaluation (SCORE) tools have been widely utilized to estimate CVD risk based on factors like age, gender, cholesterol levels, blood pressure, and smoking status [[19], [20], [21], [22]]. Nevertheless, these models may inadequately estimate CV risk in populations with complex metabolic conditions like PCOS, where non-traditional risk factors, such as hyperandrogenism, chronic inflammation, and renal dysfunction, play significant roles. Furthermore, these tools generally fail to comprehensively account for the distinctive disease processes, risk factor interactions, and higher baseline risks characteristic of individuals with chronic conditions [23,24]. For instance, the FRS has been shown to underpredicted cardiac events in patients with chronic kidney disease (CKD) and overestimates risk in populations with lower baseline cardiovascular event rates, whereas the SCORE tool tends to overestimate risk in older adults and underestimate it in high-risk ethnic groups [[25], [26], [27]]. The PREVENT model, recently developed by the American Heart Association, offers a more comprehensive approach by incorporating cardiovascular-kidney-metabolic (CKM) health metrics, including urinary albumin-to-creatinine ratio (UACR) and estimated glomerular filtration rate (eGFR), alongside traditional risk factors, such as body mass index (BMI) [28]. This model’s inclusion of these additional parameters makes it particularly relevant for assessing CVD risk in women with PCOS, where renal and metabolic dysfunctions are prevalent. Evaluating the PREVENT model’s performance in this population could enhance personalized risk stratification and inform targeted interventions to mitigate CVD morbidity and mortality. Although it has been shown that this prediction model demonstrates well discrimination and calibration in the general US population [29], little information is available regarding other populations with different ethnicities, and also including those with PCOS.

Given the limitations of traditional CV risk prediction models and the potential of the PREVENT equation to address these gaps, particularly in populations with specific risk profiles, therefore this study aimed to assess the performance of the PREVENT risk model, in terms of discrimination and calibration in the overall female sample representative of the Iranian general population, across age groups, and among women with PCOS.

## 2 Method

This study was conducted according to the guidelines of the Declaration of Helsinki, and all its procedures involving human subjects were approved by the ethics committee of the Research Institute for Endocrine Sciences, Shahid Beheshti University of Medical Sciences. Written informed consent was obtained from all subjects.

### 2.1 Study cohort

For the purpose of the current study, we used data from Tehran Lipid and Glucose Study (TLGS), a large prospective population-based cohort study, initiated in 1998 to investigate risk factors for non-communicable diseases, mainly cardio-metabolic disorders. The details of this study have been published before [30,31]. In brief, participants aged ≥3 years old were selected through multistage cluster random sampling and have been evaluated at approximately 3-year intervals through seven data collection phases.

### 2.2 Study population

For the current analysis, we utilized data from phases 3 (2006–2008) to 7 (2019- 2021) of the TLGS, which initially included 15,825 participants. Male participants (n = 6260), individuals lacking follow-up data (n = 1789) and those females who suffered from CVD at baseline (*n* = 105) were excluded from the study. Among the remaining participants, those with clinical parameters outside the validated input ranges for the PREVENT equations were also excluded, including individuals with age <30 or >80 years (n = 3204), total cholesterol <130 or >320 mg/dL (n = 121), systolic blood pressure (SBP) <90 or >200 mmHg (n = 163), body mass index (BMI) <18.5 or >39.9 kg/m² (n = 168), unknown diabetes status (n = 24), and missing data on smoking status or high-density lipoprotein (HDL) cholesterol (n = 8). Eligible women were stratified into four age groups: 30–44, 45–54, 55–64 and 65–79 years.

**Fig. 1:**
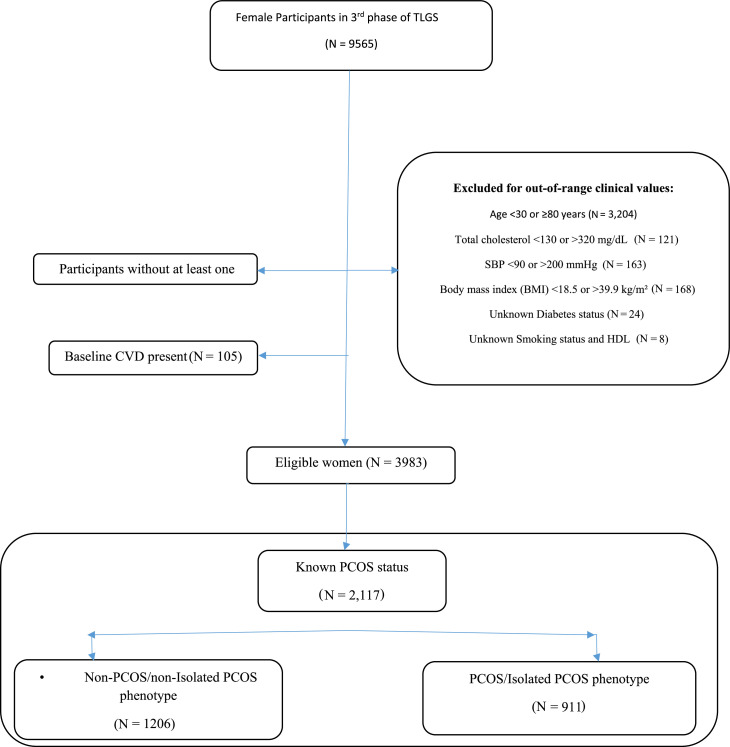
Flowchart of study. TLGS: Tehran Lipid and Glucose Study; PCOS: Polycystic ovary syndrome; CVD: Cardiovascular disease; SBP: systolic blood pressure; BMI: Body mass index; HDL-C: high-density lipoprotein- Cholesterol.

Secondary, among eligible participants, 2117 women had confirmed data on PCOS status and were further categorized into women with confirmed or isolated PCOS phenotypes (*n* = 911), and non-PCOS/Isolated PCOS phenotype controls (*n* = 1206). The PREVENT risk score equations were employed to estimate the risk of CVD among participants. A flowchart of the study population is presented in Fig. 1.

### 2.3 Clinical, biochemical and hormonal assessments

All physical examinations, laboratory assessment and interviews, including Ferriman–Gallwey hirsutism scoring, were carried out by trained personnel based on TLGS protocol. Cardiovascular risk estimation was done using American Heart Association PREVENT™ risk calculator that uses traditional risk factors such as age, blood pressure, lipid profile, diabetes, smoking, BMI, and kidney function to predict the risk of 10- year ASCVD risk [28].

Following interviews, the subjects underwent physical examinations. Height was measured using a tape stadiometer while the subject stood barefooted, with heels, buttocks, and upper back against a vertical wall and arms in a neutral shoulder position. Body weight was recorded using a calibrated electronic digital scale with participants wearing minimal clothing, (Seca 707, Hanover, MD, USA) and values rounded to the nearest 100 g. Blood pressure was measured by trained personnel using a calibrated mercury sphygmomanometer on the participant's right arm after the participant sat and relaxed for at least 15 min. The cuff size that best fitted the participant's arm circumference was used. Two measurements 60 s apart were obtained, and the average of the two was used as the final blood pressure. Systolic blood pressure (SBP) was measured at the onset of the first Korotkoff sound (Phase I), and diastolic blood pressure (DBP) at the final Korotkoff sounds fade away (Phase V), as per standard protocol. Participants provided written informed consent.

Fasting venous blood samples were collected in the early follicular phase of spontaneous or progesrton induced menstrual cycles in the morning to perform biochemical and hormonal measurements. Triglyceride (TG) levels were measured using the glycerol phosphate oxidase method. Total cholesterol (TC) levels were measured enzymatically using cholesterol esterase and cholesterol oxidase in a colorimetric assay. High-density lipoprotein cholesterol (HDL-C) was assessed after precipitation of apolipoprotein B-containing lipoproteins using phosphotungstic acid. Low-density lipoprotein cholesterol (LDL-C) was calculated according to the Friedewald formula. Serum creatinine (Cr) levels were measured using the kinetic colorimetric Jaffe method, with an assay sensitivity of 0.2 mg/dL (range: 0.2–15 mg/dL or 18–1330 μmol/L). Commercial kits (Pars Azmoon Inc., Tehran, Iran) were used for biochemical assessment by Selectra 2 autoanalyser (Vital Scientific, Spankeren, The Netherlands). The intra- and inter-assay coefficients of variation for all these biochemical assays were maintained below 3.1 % throughout the study. Dehydroepiandrosterone sulfate (DHEAS), 17‑hydroxy- progesterone (17OH-P), Total testosterone (TT) and androstendion(A4) were assessed using enzyme immu- noassay (EIA), (Diagnostic biochem canada Co. Ontario, Canada). Sex Hormone Binding Globulin (SHBG) was measured using immunoenzymometric assay (IEMA), (Mercodia, Uppsala, Sweden). All ELISA tests were accomplished using the Sunrise ELISA reader (Tecan Co. Salz- burg, Austria). The free androgen index (FAI) was computed using the formula [TT (nmol/L) × 100/SHBG (nmol/L)]. Intra-and inter-assay coefficients of variation for TT were 3.6 % and 6.0 %; for DHEAS: 1.9 % and 3.2 %; for 17 OH-P: 5.1 % and 6.2 %; for SHBG: 1.1 % and 4.1 %; for A4 they were 2.2 % and 3.5 % [1].

### 2.4 Outcome of study

The primary outcome of the present analysis was CVD events including atherosclerotic cardiovascular disease (ASCVD) and heart failure (HF) (26). Details of the collection of CVD events data have been published elsewhere [32]. In brief, participants underwent annual follow-ups conducted by trained nurses via phone calls to identify any CVD events within the past year. If any events occurred a trained physician collected the necessary data through home visits and/or by reviewing hospital records. Subsequently, the diagnosis was confirmed by the Cohort Outcome Panel of medical specialists including internist, endocrinologist, cardiologist, epidemiologist, and other experts evaluated the collected data to assign a specific outcome for every event. In the current study, all CVD events were defined as a composite measure of any cases of definite and probable myocardial infarction (MI), unstable angina, angiographic proven chronic heart disease (CHD), CHD death, definite or possible stroke, transient ischemic attack or cerebrovascular death. Hard CVD was defined as a composite of death from CVD, non-fatal myocardial infarction, or non-fatal stroke.

### 2.5 Definition of terms

PCOS was defined based on the Rotterdam criteria. Details are presented in supplementary material 1. Isolated PCOS phenotypes were defined as the presence of only one of the diagnostic features of PCOS.

Body mass index (BMI) was calculated by dividing weight in kilograms by height in meters squared (kg/m²). Smoking status was classified as ever and never smoker. A positive family history of CVD was defined as a diagnosis of CVD in a first-degree female relative before the age of 65, or in a first-degree male relative before the age of 55. The estimated glomerular filtration rate (eGFR) was calculated using the abbreviated prediction equation developed by the Modification of Diet in Renal Disease (MDRD) study, as follows: eGFR (mL/min/1.73 m²) = 186 × (serum creatinine)^−1.154^ × (age)^−0.203^ × 0.742 [33]. The PREVENT risk score equations includes traditional risk factors of age, sex, SBP, TC, HDL-C, BMI, diabetes status, smoking status, and the use of antihypertensive or statin medications and eGFR [28].

### 2.6 Statistical analysis

We assessed the normality of continuous variables using the Kolmogorov-Smirnov test. For normally distributed data, we reported the mean (standard deviation), for non-normally distributed data, we presented the median and interquartile range. Categorical variables are expressed as frequencies and percentages and compared using the chi-square test. Model discrimination was assessed using Harrell's C-statistics. Furthermore, time-dependent Area Under the Curve (AUC) was calculated to evaluate discrimination at various time points. Calibration was assessed by estimating observed 10-year CVD risk using Kaplan-Meier survival probabilities and comparing these with predicted risks. This comparison was graphically represented by plotting mean predicted risk with mean observed risk for each group. All eligible women were divided into deciles based on the PREVENT model's predicted risks. Calibration was also assessed within age groups (30–44, 45–54, 55–64, and 65–79) and by PCOS status. Because these subgroup analyses had smaller sample sizes and fewer events, predicted risks were divided into quartiles rather than deciles to improve stability.

## 3 Results

Table 1 presents baseline characteristics for all eligible women and subgroups stratified by PCOS status. The median follow-up time for 3983 eligible women was 12.23 years. The mean age and BMI of study population were 47.60 years and 28.57 kg/m², respectively. Women with PCOS/Isolated PCOS phenotype group had a significantly younger mean age (40.10 ± 6.48 vs. 42.33 ± 7.42 years, P-value <0.001), lower TC levels (189.97 ± 34.31 vs. 193.40 ± 34.29 mg/dL, P-value: 0.023), and higher estimated glomerular filtration rate (eGFR) (76.00 ± 12.38 vs. 74.60 ± 12.50 mL/min/1.73m², P-value: 0.010) compared to Non-PCOS/non- Isolated PCOS phenotype women. No statistically significant differences were observed between the two groups in HDL-C, SBP, BMI, smoking status, T2DM prevalence, or medication use.

**Table 1:** Baseline characteristics of the study population in the general female population (*n* = 3983, biological replicates) and subgroups by age (30–44 years, *n* = 1797; 45–54 years, *n* = 1065; 55–64 years, *n* = 702; 65–79 years, *n* = 419, biological replicates) and PCOS/Isolated PCOS phenotype status (Non-PCOS/non-Isolated PCOS phenotype, *n* = 1206; PCOS/Isolated PCOS phenotype, *n* = 911, biological replicates). Data are presented as mean (SD) or median [IQR] for continuous variables and number ( %) for categorical variables. P-values were calculated using one-way ANOVA for continuous variables and chi-square test for categorical variables, with statistical significance set at *P* < 0.05.

Variable		General female population (*n* = 3983)	Subgroups of age				P-value	Subgroups of PCOS/ Isolated PCOS phenotype		P-value
			30–44 years (*n* = 1797)	45–54 years (*n* = 1065)	55–64 years (*n* = 702)	65–79 years (*n* = 419)		Non-PCOS/non- Isolated PCOS phenotype (*n* = 1206)	PCOS/Isolated PCOS phenotype (*n* = 911)	
Age (years), Mean (SD)		47.60 (11.68)	37.11 (4.26)	49.23 (2.87)	59.02 (2.82)	69.37 (3.70)	<0.001	42.33 (7.42)	40.10 (6.48)	<0.001
BMI (kg/m²), Median [IQR]		28.57 [25.78, 31.99]	27.48 [24.89, 30.76]	29.37 [26.84, 32.44]	29.94 [27.05, 32.89]	28.83 [25.98, 31.99]	<0.001	28.08 [25.32, 31.24]	27.94 [25.40, 31.21]	0.862
Total-Cholesterol (mg/dL)		199.81 (36.73)	185.14 (31.64)	205.47 (35.48)	218.19 (36.63)	217.55 (34.76)	<0.001	193.40 (34.29)	189.97 (34.31)	0.023
HDL-Cholesterol (mg/dL), Median [IQR]		45.00 [38.00, 52.00]	45.00 [38.00, 52.00]	44.00 [38.00, 51.00]	44.00 [37.00, 51.00]	45.00 [39.00, 53.00]	0.139	45.00 [38.00, 51.00]	44.00 [37.00, 52.00]	0.129
SBP (mmHg), Mean (SD)		115.46 (17.72)	107.09 (11.62)	116.46 (15.82)	124.85 (18.77)	133.08 (20.48)	<0.001	110.36 (14.69)	108.94 (12.48)	0.019
eGFR (ml/min/1.73m²), Mean (SD)		70.93 (13.56)	78.00 (11.99)	69.64 (11.21)	63.26 (10.46)	56.76 (10.48)	<0.001	74.60 (12.50)	76.00 (12.38)	0.010
Ever Smoker, yes ( %		174 (4.4)	58 (3.2)	63 (5.9)	34 (4.8)	19 (4.5)	0.006	44 (3.6)	35 (3.8)	0.907
Type2 Diabetes, yes ( %)		556 (14.0)	88 (4.9)	160 (15.0)	178 (25.4)	130 (31.0)	<0.001	96 (8.0)	58 (6.4)	0.189
Medication Use, yes ( %)										
	Antihypertensive medication	264 (6.6)	25 (1.4)	61 (5.7)	99 (14.1)	79 (18.9)	<0.001	36 (3.0)	21 (2.3)	0.411
	Lipid-Lowering medication	271 (6.8)	30 (1.7)	61 (5.7)	102 (14.5)	78 (18.6)	<0.001	39 (3.2)	29 (3.2)	0.990

### 3.1 Discrimination

**Fig. 2:**
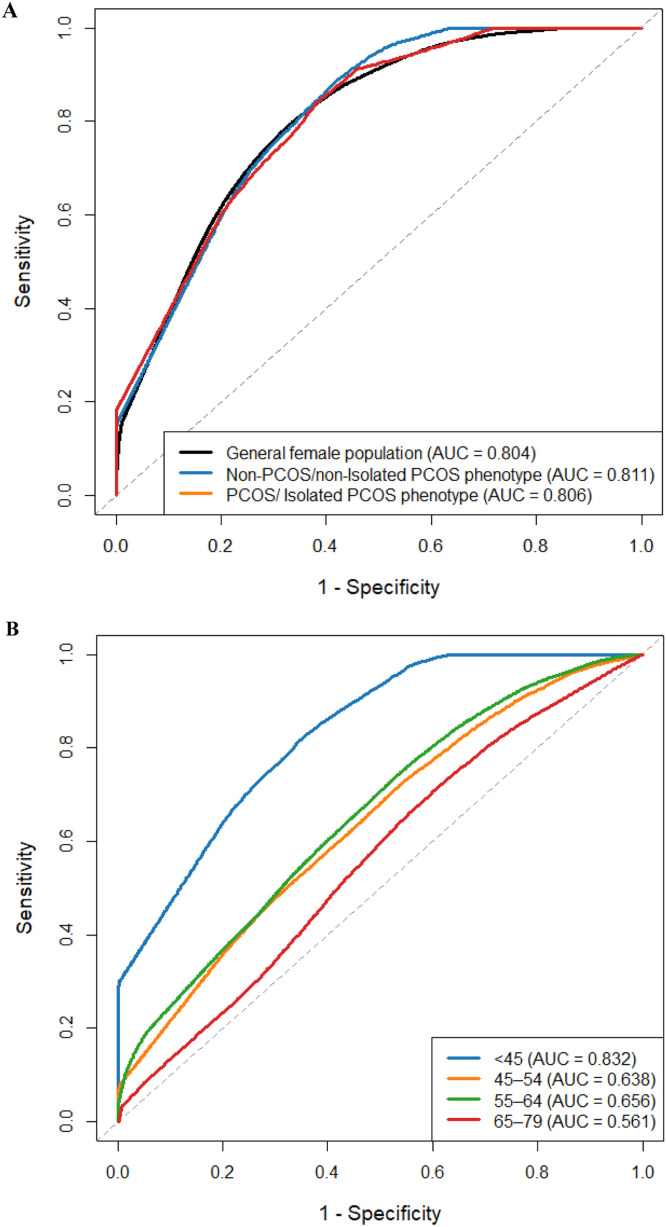
The 10-year ROC curves for (A) General female population and subgroup of PCOS/Isolated PCOS phenotype as Non-PCOS/non- Isolated PCOS phenotype (*n* = 1206) and PCOS/Isolated PCOS phenotype (*n* = 911)and (B) and by age subgroup as 30–44 years (*n* = 1797), 45–54 years (*n* = 1065), 55–64 years (*n* = 702), 65–79 years (*n* = 419) and PCOS status. P-values were calculated using DeLong’s test to compare AUCs across subgroups, with statistical significance set at *P* < 0.05.

Table 2 presents number of events, incidence rate and the discrimination performance of the PREVENT risk model using Harrell’s C-statistics and AUC values with their 95 % confidence intervals and Fig. 2 (A and B) illustrates the 10-year ROC curves for all eligible female participants, stratified by age subgroups and PCOS status.

**Table 2:** Incidence rates of CVD events and discrimination performance of the risk prediction model by subgroups of age, as 30–44 years (*n* = 1797), 45–54 years (*n* = 1065), 55–64 years (*n* = 702), 65–79 years (*n* = 419) and PCOS status as Non-PCOS/non- Isolated PCOS phenotype (*n* = 1206) and PCOS/Isolated PCOS phenotype (*n* = 911); PCOS = polycystic ovary syndrome, AUC: Area Under the Curve. Incidence rates were calculated as the number of incident CVD events per 1000 person-years of follow-up. Harrell’s C-statistics and AUC (95 % CI) are reported for model discrimination. P-values were derived from DeLong’s test for comparing AUCs across subgroups, with statistical significance set at *P* < 0.05.

Group		N	Events, n ( %)	Person-years	Incidence rate (per 1000 Person-Year)	Harrell’s C-statistics (95 % CI)	AUC (95 % CI)
All eligible female participants		3983	378 (9.5)	44,047.5	8.58	0.84 (0.82–0.86)	0.80 (0.77–0.83)
Age subgroups							
	Age 30–44 years	1797	24 (1.3)	20,747.1	1.16	0.82 (0.75–0.89)	0.83 (0.72–0.92)
	Age 45–54 years	1065	80 (7.5)	12,067.1	6.63	0.71 (0.65–0.77)	0.64 (0.59–0.72)
	Age 55–64 years	702	127 (18)	7441.1	17.07	0.67 (0.62–0.72)	0.66 (0.61–0.71)
	Age 65–79 years	419	147 (35)	3792.1	38.76	0.58 (0.53–0.63)	0.56 (0.52–0.61)
PCOS/ Isolated PCOS phenotype subgroups							
	PCOS/ Isolated PCOS phenotype	911	21 (2.3)	11,128.0	1.89	0.79 (0.71–0.87)	0.81 (0.74–0.94)
	Non-PCOS/non- Isolated PCOS phenotype	1206	41 (3.4)	14,520.3	2.82	0.85 (0.80–0.89)	0.81 (0.75–0.87)

PCOS: polycystic ovary syndrome; AUC: Area Under Curve.

During 44,047.5 person-years of follow-up, 378 incident CVD events were observed among overall cohort of female participants, corresponding to an overall incidence rate of 8.58 per 1000 person-years. CVD incidence increased significantly with advancing age, from 1.16 per 1000 person-years in women aged 30–44 years to 6.63, 17.07, and 38.76 per 1000 person-years in the 45–54, 55–64, and 65–79 year age groups, respectively. By PCOS status, women with PCOS/isolated PCOS phenotype exhibited a lower CVD incidence rate (1.89 per 1000 person-years) compared with those without PCOS/isolated PCOS phenotype (2.82 per 1000 person-years).

Among women aged <55 years, the incidence rate of ASCVD/CVD was 1.82 per 1000 person-years in those with PCOS/ Isolated PCOS phenotype and 2.55 per 1000 person-years in Non-PCOS/non- Isolated PCOS phenotype women, corresponding to an incidence rate ratio of 0.71 (95 % CI 0.41–1.23) (Supplementary Table 1).

Among all female participants, the model demonstrated good discrimination, with a Harrell’s C-statistic of 0.84 (95 % CI: 0.82–0.86) and an AUC of 0.80 (95 % CI: 0.77–0.83) (Table 2, Fig. 2-A). Discriminatory ability was similarly high among subgroup of women with and without PCOS/Isolated PCOS phenotype (C-statistic 0.79, 95 % CI: 0.71–0.87; AUC 0.81, 95 % CI: 0.74–0.94) and (C-statistic 0.85, 95 % CI: 0.80–0.89; AUC 0.81, 95 % CI: 0.75–0.87), respectively (Table 2, Fig. 2-A). Subgroup analysis by age showed that discrimination declined with increasing age (Table 2, Fig. 2-B). The model performed best in women aged 30–44 years (C-statistic 0.82, 95 % CI: 0.75–0.89; AUC 0.83, 95 % CI: 0.72–0.92), but performance progressively weakened in older groups.

### 3.2 Calibration

**Fig. 3:**
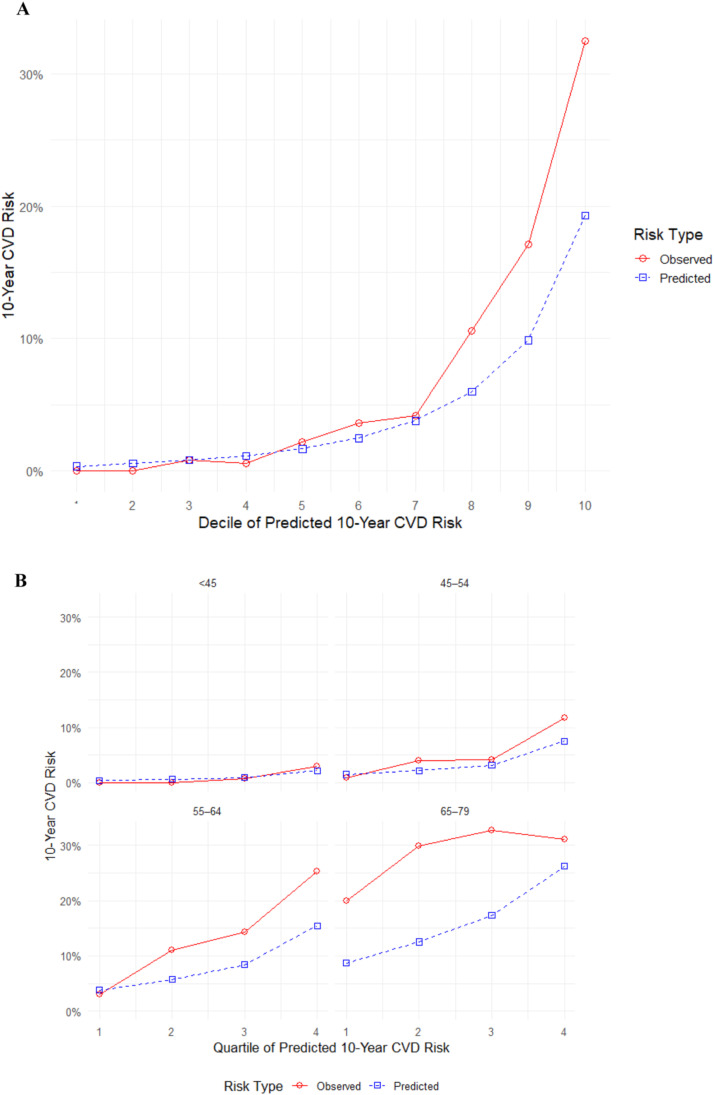

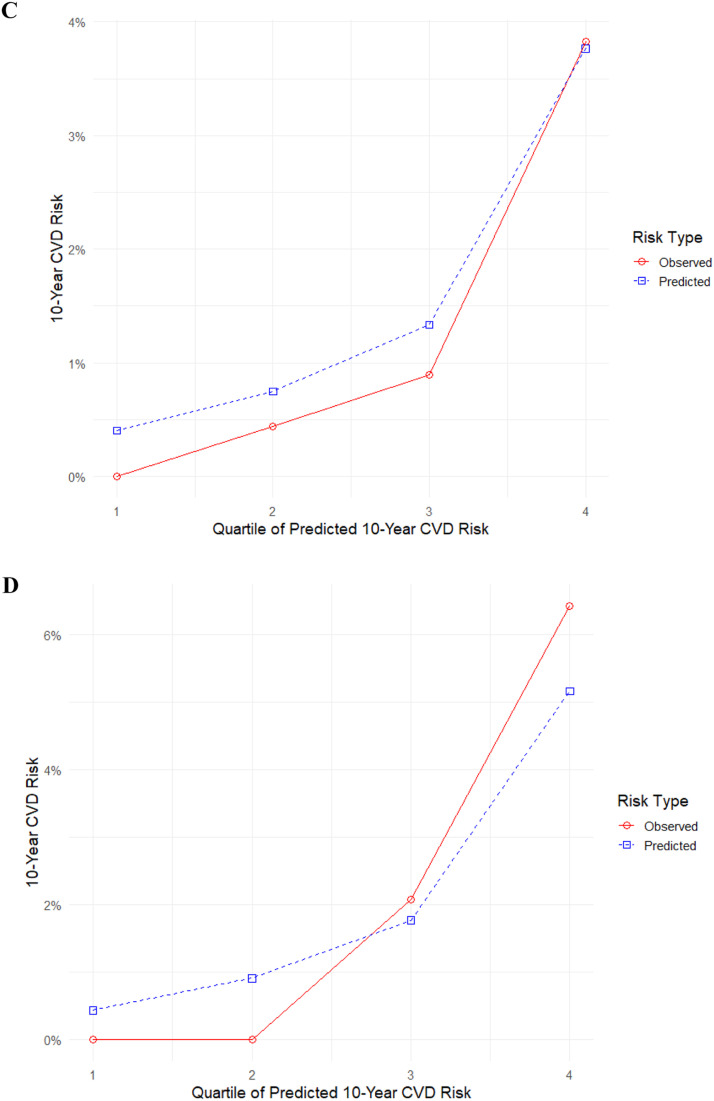
Calibration of the PREVENT model. (A) General female population (*n* = 3983) (B) By age subgroups as 30–44 years (*n* = 1797), 45–54 years (*n* = 1065), 55–64 years (*n* = 702), 65–79 years (*n* = 419) (C) Females with PCOS or isolated PCOS phenotype (*n* = 911). (D) Females without PCOS or isolated PCOS phenotype (*n* = 1206). Numeric group labels represent quantiles of predicted 10-year CVD risk (quartiles or deciles, as applicable), ordered from lowest to highest risk. P-values were derived from the Hosmer-Lemeshow test to assess calibration goodness-of-fit, with statistical significance set at *P* < 0.05.

Fig. 3 displays calibration of the PREVENT model. Among general female population, predicted and observed risks were closely aligned in the lower and mid-risk deciles, indicating satisfactory calibration in these groups. However, in the highest deciles, the model consistently underestimated 10-year CVD risk, with the discrepancy most pronounced among women at the upper end of the predicted risk spectrum (Fig. 3-A). Age-stratified calibration analyses revealed accurate agreement between predicted and observed risks for women younger than 55 years in subgroups of > 45 years and 45–54 years. The model underestimated observed CVD risk for women in the higher quartiles, those between the ages of 55–64 and 65–79, with the greatest miscalibration observed in women aged 65–79 years (Fig. 3-B). Calibration curves for women with PCOS or isolated PCOS phenotype and women without PCOS or isolated PCOS phenotype are displayed in (Fig. 3-C) and (Fig. 3-D), respectively. Due to variations caused by small sample sizes and few events, the model overestimates risk in some deciles while underestimating it in others. Overall, calibration remained satisfactory in both groups.

## 4 Discussion

In this large population-based cohort of Iranian women followed for over two decades, the American Heart Association PREVENT risk model demonstrated good overall discrimination and acceptable calibration for predicting 10-year CVD risk among both the general female population and women with PCOS/Isolated PCOS phenotypes. However, discriminatory ability declined progressively with increasing age, and calibration showed systematic underestimation of risk in higher-risk deciles, particularly among women aged 55 years and older.

Cardiovascular disease is the leading global cause of death, responsible for about 19.8 million deaths in 2022, and imposes a substantial economic burden on healthcare systems [34,35]. Clinical guidelines for preventing CVD often recommend the use of risk scores or prediction models that evaluate the combined effect of multiple measured risk factors [[36], [37], [38]]. These tools estimate an individual’s future risk of CVD and help identify those who are most likely to benefit from preventive measures [[36], [37], [38], [39]]. Today, several CV risk prediction models are available for clinical use, including Framingham Risk Score, Pooled Cohort Equations, Systematic Coronary Risk Evaluation (SCORE and SCORE2), WHO cardiovascular risk charts, PROCAM, PREDICT, and QRISK3 [[19], [20], [21], [22],^40^,^41^]. Despite their widespread application, these models exhibit important limitations. The FRS was derived from a predominantly white, middle-class community and lacks generalizability to more diverse populations. It estimates only CHD risk, excluding other major outcomes like stroke and heart failure [42]. Furthermore, the FRS also performs poorly in individuals with metabolic disorders, leading to risk underestimation of risk in Individual with diabetes or related conditions [43]. Additionally, both the FRS and PROCAM underestimate CHD risk in women and younger individuals, thereby compromising accuracy in sex- and age-specific prediction [44]. The PCE, while more broadly validated, is based on older cohorts, which may lead to overestimation of atherosclerotic CVD (AS-CVD) risk due to shifts in risk factor prevalence, such as smoking and cholesterol levels [45]. It also excludes non-ASCVD outcomes such as heart failure and may overstate risk in older adults [46]. SCORE and SCORE2, widely used in Europe, are primarily designed to predict fatal CVD events and may therefore underestimate the burden of non-fatal but clinically significant outcomes, limiting their utility for prevention in younger women [22]. Similarly, although QRISK3 and PREDICT include a broader range of risk factors and show improved calibration, their generalizability is limited, QRISK3 is largely UK-specific and may not perform well in other healthcare settings, while PREDICT is derived from New Zealand cohorts and lacks validation in external populations [47]. All still lack systematic validation in women with PCOS, a group at elevated risk for type 2 diabetes, hypertension, dyslipidemia, and CVD.

The newly developed PREVENT risk model by the American Heart Association was designed to address many of shortcomings of earlier tools by incorporating contemporary cohorts, diverse populations, sex-specific, race-independent estimates of 10- and 30-year total CVD risk, and a broader spectrum of outcomes, including AS-CVD, heart failure and atrial fibrillation among adults aged 30 to 79. Unlike prior models, PREVENT uniquely integrates kidney function, measured by estimated eGFR, and account for competing risks such as non-CVD mortality. The inclusion of key chronic kidney and metabolic markers improves predictive accuracy, aligning with growing evidence that kidney function is a strong determinant of CVD. PREVENT relies on routinely available clinical variables, including age, sex, SBP, total and HDL-C, BMI, smoking status, antihypertensive and statin therapy, T2DM status, and eGFR, making it particularly relevant for individuals with impaired cardio-renal-metabolic health. In addition, it uses age as the underlying time scale rather than calendar time, providing a more accurate representation of the natural progression of CVD [28,48,49]. However, its performance in high-risk subgroups such as women with PCOS has not been evaluated.

Women with PCOS have a well-documented excess burden of cardio-metabolic risk factors [[50], [51], [52], [53]]. However, despite the clear presence of multiple CV risk factors early in life among in women with PCOS, there is still uncertainty about whether this translates into a higher incidence of CVD later in life [50,51]. Additionally, studies examining the association between PCOS on the risk of developing CVD have yielded conflicting results, largely due to methodological limitations such as retrospective designs, inclusion of women beyond menopause without clear evidence of PCOS during the reproductive period, overrepresentation of more severe phenotypes and lack of adjustment for main potential confounders. Consequently, the long-term impact of PCOS on CVD risk, particularly in late reproductive and postmenopausal women is still unclear [52,54]. To date, no CV risk prediction model has been specifically developed or validated for this population. Our previous study demonstrated that the Framingham Risk Score is applicable to women with PCOS, showing a 38 % increase in CVD risk for each one-unit rise in the FRS [52]. Similarly, Meun et al. utilized the FRS in middle-aged women with PCOS and reported despite a moderately adverse cardiometabolic profile, these women did not exhibit higher 10-year CVD risk or greater atherosclerosis compared with age-matched controls [55]. However, we hypothesized that the PREVENT risk equations may be particularly well-suited for CVD assessment in women with PCOS, given the adverse impact of PCOS on renal function. By integrating these parameters, the model may enhance predictive accuracy and enable more personalized risk stratification.

In the present study, the risk prediction model demonstrated the highest calibration accuracy among individuals younger than 45 years, with performance declining progressively with age. This pattern may be attributed to the more homogeneous CV risk profiles and lower prevalence of confounding comorbidities in younger populations, which reduce model complexity and enhance predictive accuracy. In contrast, models exhibit less effectively in older groups, as restricting the age range reduces the variability in the most influential predictor, age itself [28,56]. The discrepancy observed between predicted and observed CV events in older participants may reflect contextual factors; specifically, the elderly individuals in this cohort lived during a period when routine CV screening and preventive or therapeutic interventions were uncommon in our country, whereas the models were developed and validated in populations with long-established, widely implemented screening programs. Consequently, the observed incidence CVD in older adults exceeded the model-based predictions.

This study has several notable strengths. To our knowledge, it is the first to evaluate the predictive performance of the PREVENT risk score for CVD specifically among women with PCOS. The prospective design and inclusion of an unselected population and extended follow-up exceeding twenty years enhance the robustness and generalizability of our findings within this cohort. Nonetheless, some limitations should be considered. The overall number of CVD events observed was relatively low; however, the statistical power remained adequate to yield precise risk estimates for the general population. In contrast, the PCOS subgroup experienced a limited number of events, which constrained the reliability of the model's performance evaluation within this subgroup. Consequently, the findings related to women with PCOS should be interpreted with caution due to this limited event count. As in many epidemiological studies, eGFR was defined based on single creatinine measurements, which are subject to daily variability and were not confirmed by repeat assessments over three months, potentially affecting the accuracy of measurement. Additionally, the sample size was insufficient to evaluate CVD risk across distinct clinical phenotypes of PCOS. Finally, as this study exclusively involved Iranian women, caution is warranted when generalizing the findings to populations with differing demographic and clinical characteristics.

## 5 Conclusion

In conclusion, in this long-term, population-based cohort of Iranian women, the PREVENT risk model demonstrated good discrimination and acceptable calibration for predicting 10-year CVD risk in both the general female population and women with PCOS. The model performed best in younger women (<55 years), while discrimination and calibration declined in older age groups, with systematic underestimation of risk in higher-risk deciles, particularly among those aged 55 years and above. These findings support the utility of the PREVENT model for CVD risk assessment in women, including those with PCOS, while the need for caution when interpreting predictions in older or high-risk individuals.

## Data statement

Data was obtained from the Tehran lipid and glucose study. TLGS data will be shared on reasonable requests to the corresponding author agreement.

## Sources of funding

This study funded by the 10.13039/501100007427Research Institute for Endocrine Sciences, Shahid Beheshti University of Medical Sciences, Tehran, Iran (Grant number: 4-43017510). Nord University covered the article processing charge for the publication.

## CRediT authorship contribution statement

**Parham Heidari:** Writing – original draft, Data curation, Conceptualization. **Ramin Farrokhi:** Writing – review & editing, Methodology, Formal analysis. **Faegheh Firouzi:** Writing – original draft, Investigation, Data curation. **Yasamin Zivari:** Writing – review & editing, Investigation. **Fereidoun Azizi:** Writing – review & editing, Data curation. **Fahimeh Ramezani Tehrani:** Writing – review & editing, Methodology, Formal analysis, Data curation, Conceptualization. **Samira Behboudi-Gandevani:** Writing – original draft, Supervision, Investigation, Funding acquisition, Conceptualization.

## Declaration of competing interest

The authors declare the following financial interests/personal relationships which may be considered as potential competing interests:

Samira Behboudi-Gandevani reports article publishing charges was provided by Nord University. If there are other authors, they declare that they have no known competing financial interests or personal relationships that could have appeared to influence the work reported in this paper.

## Appendix Supplementary materials

Image, application 1
