# Supplementary material for: Prediction of cardiovascular disease risk in women and individuals with polycystic ovary syndrome using the American Heart Association PREVENT model: A long-term population-based cohort study
Source: Am J Prev Cardiol. 2026 Jan 3;25:101408. doi: 10.1016/j.ajpc.2026.101408 (PMC12848994; doi:10.1016/j.ajpc.2026.101408)
Supplement: Supplementary file 1 [file mmc1.docx]

Supplementay material

Supplementary material 1:

PCOS was defined based on the Rotterdam criteria, including presence of at least two of three criteria as follows: oligo/anovulation, clinical or biochemical hyperandrogenism, and polycystic ovarian morphology (PCOM) on ultrasound. Oligo/anovulation was defined as having either regular or irregular menstrual cycles lasting 34 days or longer, or a history of eight or fewer menstrual cycles per year. Clinical signs of hyperandrogenism included hirsutism, assessed using the modified Ferriman–Gallwey scoring system using a cutoff score of ≥8, as well as acne and androgenic alopecia. Biochemical hyperandrogenism Biochemical hyperandrogenism was defined as elevated serum levels of one or more androgens including dehydroepiandrosterone sulfate (DHEAS), total testosterone (TT), free androgen index (FAI), or androstenedione (A4), exceeding the 95th percentile thresholds established from a reference population of healthy, non-hirsute, eumenorrheic women. The corresponding cutoff values were TT > 0.89 ng/mL, A4 > 2.9 ng/mL, DHEAS > 179 μg/dL, and FAI > 5.39 [1]. PCOM was defined by the presence of ≥12 follicles measuring 2–9 mm in diameter in each ovary and/or an ovarian volume >10 cm³ [1].

**Reference**

1. Farhadi-Azar, M., et al., The Prevalence of Polycystic Ovary Syndrome, Its Phenotypes and Cardio-Metabolic Features in a Community Sample of Iranian Population: Tehran Lipid and Glucose Study. Front Endocrinol (Lausanne), 2022. 13: p. 825528

Supplementary Table 1. Age-restricted incidence of ASCVD/CVD events (<55 years) by PCOS status

| Age group | PCOS status | N | Events | Person-years | Incidence rate (per 1,000 PY) | IRR (PCOS / Isolated PCOS phenotype vs Non-PCOS/non- Isolated PCOS phenotype), 95% CI |
| --- | --- | --- | --- | --- | --- | --- |
| <55 years | Non-PCOS/non- Isolated PCOS phenotype | 1,165 | 36 | 14,102 | 2.55 | Reference |
| <55 years | PCOS / Isolated PCOS phenotype | 898 | 20 | 10,998 | 1.82 | 0.71 (0.41–1.23) |
